# Barriers to effective discharge planning: a qualitative study investigating the perspectives of frontline healthcare professionals

**DOI:** 10.1186/1472-6963-11-242

**Published:** 2011-09-29

**Authors:** Eliza LY Wong, Carrie HK Yam, Annie WL Cheung, Michael CM Leung, Frank WK Chan, Fiona YY Wong, Eng-Kiong Yeoh

**Affiliations:** 1 Division of Health Systems, Policy and Management, School of Public Health and Primary Care, The Chinese University of Hong Kong, Hong Kong

## Abstract

**Background:**

Studies have shown that effective discharge planning is one of the key factors related to the quality of inpatient care and unnecessary hospital readmission. The perception and understanding of hospital discharge by health professionals is important in developing effective discharge planning. The aims of this present study were to explore the perceived quality of current hospital discharge from the perspective of health service providers and to identify barriers to effective discharge planning in Hong Kong.

**Methods:**

Focus groups interviews were conducted with different healthcare professionals who were currently responsible for coordinating the discharge planning process in the public hospitals. The discussion covered three main areas: current practice on hospital discharge, barriers to effective hospital discharge, and suggested structures and process for an effective discharge planning system.

**Results:**

Participants highlighted that there was no standardized hospital-wide discharge planning and policy-driven approach in public health sector in Hong Kong. Potential barriers included lack of standardized policy-driven discharge planning program, and lack of communication and coordination among different health service providers and patients in both acute and sub-acute care provisions which were identified as mainly systemic issues. Improving the quality of hospital discharge was suggested, including a multidisciplinary approach with clearly identified roles among healthcare professionals. Enhancement of health professionals' communication skills and knowledge of patient psychosocial needs were also suggested.

**Conclusions:**

A systematic approach to develop the structure and key processes of the discharge planning system is critical in ensuring the quality of care and maximizing organization effectiveness. In this study, important views on barriers experienced in hospital discharge were provided. Suggestions for building a comprehensive, system-wide, and policy-driven discharge planning process with clearly identified staff roles were raised. Communication and coordination across various healthcare parties and provisions were also suggested to be a key focus.

## Background

Hospital discharge is a complex and challenging process for healthcare professionals, patients, and carers. Effective discharge planning could significantly improve a patient's health and reduce patient readmission [1-6]. A systematic review from 21 randomised controlled trials involving 7,234 patients by Shepperd showed that a structured discharge plan tailored to the individual patient probably brings about small reductions in hospital length of stay and readmission rates for older people admitted to hospital with a medical condition [7]. Studies in the United States (US) showed that increased readmissions may reflect the following: sub-optimal assessment of readiness for discharge, fragmented discharge planning, a breakdown in communication and information transfer between hospital-based and community physicians, inadequate post-discharge care and follow-up, or some combination of these processes [8-16]. The resolution may require better care coordination or a comprehensive discharge planning system [14-16].

In view of the importance of an effective discharge planning system in both acute and sub-acute care policy and practice, many countries have launched a series of guidelines for good practices in hospital discharge planning process. In the United Kingdom (UK), the National Health Services (NHS) Plan included a commitment to ensure that by 2004, every NHS patient should have a discharge plan starting from hospital admission. The Department of Health's guidance for England also said that discharge planning from a hospital is a process, instead of an isolated event, which should start at the earliest opportunity [17,18]. Effective discharge has also been a priority area in Australia since 1998. The Victoria Government has set an "Effective Discharge Strategy," a five-year initiative from 1998/99-2002/03 for all Victorian public hospitals. This initiative aimed to encourage healthcare providers to review and improve transitioning processes and practices, develop and implement performance indicators to measure the effectiveness of discharge, and reward hospitals with good practices in the transitioning of patients [19]. In the US, discharge planning is a legally mandated function for hospitals. It is also one of the "basic" hospital functions as outlined in Medicare's Conditions of Participation from Centres for Medicare & Medicaid Services. The need to establish an effective discharge planning policy and guideline has been given attention, together with the trend toward starting the discharge planning process upon admission, adopting a multidisciplinary approach, and coordinating for post-discharge care support [20]. In Hong Kong, the discharge planning policy is conducted on a piecemeal basis and is only initiated by physicians. A system-wide discharge planning policy has not been established in Hong Kong. Thus, this study would explore the barriers inhibiting effective discharge and the efficient components of discharge planning so as to provide important information for developing an effective discharge planning system. This present study seeks to extend the findings of previous studies on avoidable readmission, which highlights the need for having effective discharge planning [21,22].

In Hong Kong, about 90% of hospital-based acute care and rehabilitation services are provided by the Hong Kong Hospital Authority (HA), an independent public sector organization. The expense of healthcare financing relies on general taxation. Demand for hospital beds increased due to the arising need of an ageing population and the chronic disease burden. The pressure for more beds in acute care hospitals causes the transfer of an increasing number of patients to convalescent and rehabilitation hospitals. Alternately, patients are discharged home with ambulatory and community care after their acute illness has stabilized, even though they still require medical treatment, rehabilitation, and nursing care at the sub-acute level. Our previous study showed that the overall 30-day unplanned readmission rate was 16.7% in 2007 [23]. Our another study further highlighted the need for an effective discharge planning system to reduce avoidable hospital readmissions [21,22]. Building a comprehensive discharge planning system requires a framework to identify the differing patient needs and the appropriate services among what is available. A systematic approach through extensive literature review and inputs from key stakeholders in healthcare to develop the structure and key processes of the discharge planning system is critical to ensure not only the quality of care, but also to maximize organizational effectiveness. Today, there are mainly two different purposes underpinning discharge planning. One is to transfer both care and budgetary responsibility from the hospital to other agencies for patients who are not in need of in-patient care. Another purpose is to plan for patients' continuing health and social care [24].

This present study aims to identify current discharge planning practices of health professionals working in acute and rehabilitation hospitals, determine the barriers in executing the discharge planning of the existing system, and suggest components in developing an effective patient discharge planning system. The findings will provide information critical for the development of a discharge planning policy in Hong Kong. The present study will also provide additional information for further research into this important issue. In the next step of our study, we will be exploring the views of patients from acute and sub-acute on the discharge issue so as to provide a full picture for the development of discharge planning system within a whole-of-system perspective.

## Methods

### Participants

The qualitative method of a focus group discussion (FGD) was used to understand barriers to discharge planning and explore potential important components of effective discharge planning policy from the perspective of frontline healthcare professionals under public settings. Content analysis was used for data analysis because this emphasized the conceptual meaning and experience sharing of participants' expression [25]. Acute and rehabilitation hospitals in three HA clusters which had the highest, middle, and lowest unplanned readmission rates in 2009 were chosen. Professionals working for at least 10 years in the medical department of these hospitals, since they are most likely to understand the system, and provided valuable advice, were invited to participate in the discussion via the facilitation of HA. Eligible participants were approached for written consent. No age or sex limitation was applied to the study. At least two focus groups were held in each cluster, and the group discussion continued until issues were felt to be theoretically saturated and no new relevant data seemed to emerge [26].

### Discussion Guide

To ensure an in-depth discussion of the issues, a guided set of open-ended questions was developed based on literature review and expert opinion. The questions covered the following: (i) current practice on patient discharge planning, (ii) barriers encountered in implementing an effective patient discharge plan/program, and (iii) suggested structures and processes on an effective and comprehensive patient discharge planning system. Participants were encouraged to discuss and express their point of view.

### Data Collection Procedure

The semi-structured group discussions were conducted at the hospitals after work hours. The discussion took approximately 90-120 minutes, and the proceedings were audio-recorded with the participants' consent. The discussion was led by either ELY Wong or FWK Chan, both of them have medical backgrounds and are senior researchers in the team. Participants were allowed to freely express their views regarding the discussion topic. The discussion guide was only used to prompt questions and to ensure that the three main areas of the study were covered. The demographics of the participants, including age, gender, type of profession, and years of professional experience, were collected.

### Data Analysis

All interviews were transcribed and coded using NVivO 7.0. A mixed method of thematic analysis and grounded theory was used. Thematic analysis was first performed and themes were identified according to current practices on patient discharge planning, barriers encountered in implementing an effective patient discharge plan/program, and suggested structures and processes for an effective and comprehensive patient discharge planning system. Under the theme "barriers encountered in implementing an effective patient discharge plan/program", grounded theory was applied to categorize the barriers into 4 aspects: system, healthcare professionals, patients, and social [21,27,28]. The coding process and theme identification were independently carried out by two researchers. During the analysis, data within themes were scrutinized to disconfirm or confirm views across the range of participants. To ensure data saturation, data analysis was performed upon each completed focus group discussion to help determine whether the next focus group was needed.

### Ethical Approval

Ethical approval from the Clinical Research Ethics Committee of the Hospital Authority was obtained for the present study. Written informed consent was obtained from all participants prior to the discussion. The purpose of the present study and the right to withdraw from the focus group were explained. All discussions were recorded as anonymous and kept confidential.

## Results

In total, six sessions of FGD with different healthcare professionals from the three selected clusters were conducted in July-August 2010. A total of 41 healthcare professionals (9 Physicians, 13 Nurses, 6 Occupational Therapists, 5 Physiotherapists, and 8 Medical Social Workers) participated in the FGD. Majority of the participants were female, and the age range was 30-59 years old. The working experiences of each healthcare professional are shown in Table 1.

**Table 1:** Characteristics of Participants

Code	Interview Date	Profession (Work Setting)*	Work Setting	HA cluster^#^	Age	Gender	Work Experiences (Years)
1A	6 July, 2010	Doctor	Hospital	A	35-39	Male	15-19
1B	6 July, 2010	OT	Community	A	40-44	Female	15-19
1C	6 July, 2010	PT	Community	A	35-39	Female	10-14
1D	6 July, 2010	Geriatric Nurse	Hospital	A	40-44	Female	20-24
1E	6 July, 2010	CNS	Community	A	45-49	Male	25-29
1F	6 July, 2010	MSW	Hospital	A	35-39	Female	10-14
1G	6 July, 2010	Doctor	Hospital	A	40-44	Male	15-19
1H	6 July, 2010	Doctor	Hospital	A	45-49	Male	20-24
2A	16 July, 2010	MSW	Hospital	B	35-39	Female	10-14
2B	16 July, 2010	MSW	Hospital	B	45-49	Female	15-19
2C	16 July, 2010	CNS	Community	B	35-39	Female	10-14
2D	16 July, 2010	Doctor	Hospital	B	35-39	Male	10-14
2E	16 July, 2010	OT	Community	B	35-39	Female	15-19
2F	16 July, 2010	Geriatric Nurse	Hospital	B	35-39	Female	10-14
2G	16 July, 2010	PT	Hospital	B	30-34	Female	10-14
2H	16 July, 2010	Doctor	Hospital	B	30-34	Female	10-14
2I	16 July, 2010	Nurse	Hospital	B	45-49	Female	20-24
3A	20 July, 2010	Nurse	Hospital	A	35-39	Female	/
3B	20 July, 2010	MSW	Hospital	A	35-39	Female	15-19
3C	20 July, 2010	OT	Hospital	A	35-39	Female	15-19
3D	20 July, 2010	Doctor	Hospital	A	40-44	Female	15-19
3E	20 July, 2010	Nurse	Hospital	A	35-39	Female	20-24
3F	20 July, 2010	Geriatric Nurse	Hospital	A	55-59	Female	35-39
3G	20 July, 2010	Doctor	Hospital	A	35-39	Female	10-14
3H	20 July, 2010	PT	Hospital	A	40-44	Female	20-24
4A	22 July, 2010	MSW	Hospital	B	35-39	Female	15-19
4B	22 July, 2010	OT	Hospital	B	35-39	Female	15-19
4C	22 July, 2010	Nurse	Hospital	B	45-49	Female	15-19
4E	22 July, 2010	Doctor	Hospital	B	35-39	Female	10-14
5A	29 July, 2010	MSW	Hospital	C	35-39	Female	15-19
5B	29 July, 2010	Geriatric Nurse	Hospital	C	35-39	Female	10-14
5C	29 July, 2010	Nurse	Hospital	C	45-49	Female	20-24
5E	29 July, 2010	OT	Community	C	45-49	Female	20-24
5F	29 July, 2010	PT	Community	C	35-39	Male	15-19
6A	5 August, 2010	Doctor	Hospital	C	40-44	Female	15-19
6B	5 August, 2010	MSW	Hospital	C	45-49	Female	20-24
6C	5 August, 2010	MSW	Hospital	C	45-49	Female	10-14
6D	5 August, 2010	Nurse	Hospital	C	50-54	Female	30-34
6E	5 August, 2010	CNS	Community	C	55-59	Female	35-39
6F	5 August, 2010	PT	Hospital	C	45-49	Female	20-24
6G	5 August, 2010	OT	Hospital	C	/	Female	20-24

* OT = Occupational Therapist; PT = Physiotherapist; MSW = Medical Social Worker; CNS = Community Nurse

# A = Cluster with middle unplanned readmission rates

B = Cluster with the highest unplanned readmission rates

C = Cluster with the lowest unplanned readmission rates

The central theme in this study concludes that there was no policy-driven discharge planning with proactive and multidisciplinary approach led by the executive level in current practice. The need to establish a systematic discharge planning with standardized protocol was thus highlighted. From this central theme, two core sub-themes emerged: (i) barriers to discharge planning, and (ii) suggestions on the important components for effective discharge planning.

### Theme 1: Barriers to Discharge Planning

Participants pointed out that there were many piecemeal discharge programs in different hospitals. For example, some hospitals had disease-specific discharge programs targeting chronic obstructive pulmonary diseases, stroke, cancer, cardiovascular disease, and kidney failure. Other hospitals launched an Integrated Discharge Support Program (IDSP) for the elderly aged 65 or above. The Hospital Admission Risk Reduction Program for the Elderly (HARRPE) score was used to screen high-risk hospital readmission patients who would be recruited to the IDSP in selected hospitals. A nurse would visit the patients within 24 hours upon discharge followed up by visits from a physiotherapist, an occupational therapist, and medical social workers if necessary. Telephone nursing consultation service, liaison nurse for post-discharge service, and one-estate-one-nurse were other examples. Most participants identified a number of barriers to discharge planning, which were broadly described: system, clinician/healthcare professional, patient, and social factor [21].

#### 1.1 System Factor

Most participants expressed the system barrier as one of the major inhibitors to discharge planning. A few participants pointed out that premature discharge was due to the limited number of beds in the hospital. Owing to this pressure, some patients had very short hospital stays and were discharged too early:

"Since it is impossible to add extra beds, the length of stay must be short. Thus, there is a problem in having good discharge planning; (some patients) have to be discharged three days after admission." (6D, Nurse)

"The turnover rate and caseload in acute wards are very high... so, physicians have no time to discuss the discharge plan with the patients or their carers in detail." (6E, Community Nurse)

Participants also expressed there was some policy issues, including lack of guidelines or policies for the standard care pathway, inflexible IDSP program policy, poor medication system, and poor regulation of old age home quality:

"The care pathway is available, but it is led by the physician... If the physician does not initiate... (we will miss the patient)... Thus, if there is a policy/guideline, if the physician misses it, nurses will pick it up..." (3D, Doctor)

"We see the patient is still high risk for readmission... upon referral... they said the IDSP program cannot recruit the patient because the case is closed." (4C, Nurse)

"For changing the medication dosage, the system cannot facilitate... even though we talk to the pharmacist .... cannot change... For example, the patient is on twice daily before admission, now he is on once daily upon discharge, so the patient will have excess medication at home (but it is impossible to only print out the new medication dosage label)..." (6E, Community Nurse)

"The quality of old age home is varied...which may reflect the poor regulation..." (2B, Medical Social Worker)

Furthermore, some participants mentioned that discharge planning was a challenging task due to manpower shortage and heavy administrative work:

"Even if we have a clear clinical pathway and many pre/post discharge programs... we don't have enough manpower to follow or conduct... we already have many cases..."(6G, Occupational Therapist)

"They (physicians) have many administrative work... as a middle-level manager, we should support them, not only care for their clinical work but also support their involvement in discharge plan." (4E, Doctor)

In addition, majority of participants highlighted that communication and record transparency among healthcare disciplines were poor:

"We don't have a formal face-to-face communication... we all communicate through the chart recording."(6F, Physiotherapist)

"Communication is one-way... we only refer from acute to rehab, followed by MSW, placement problem, short course of in-patient rehab, and then home..." (3G, Doctor)

#### 1.2 Healthcare Professional Factor

The healthcare professional factor is another identified barrier. Some participants pointed out that community nurses were not empowered to involve discharge planning:

"We (community nurses) are passive... we would like to be involved in ward round or case conference, we can give advice on whether the case is ready to be discharged or not." (6E, Community Nurse)

Participants also mentioned that physicians' documents were unclear and their assessment was incomplete:

"Sometimes, we want to contact the physician but he has rotated to another hospital... or he does not have enough information due to an incomplete assessment." (6A, Doctor)

Participants also highlighted the low awareness of physicians and nurses on patient's social needs:

"We don't worry (about) the clinical part because the physician cares for it... but for the patient's social need, the physician is not mature enough to do so..." (4E, Doctor)

#### 1.3 Patient Factor

Participants further highlighted patient factor as barrier to effective discharge planning. They pointed out that the patient has a lack of knowledge of medication treatment:

"The biggest problem is medication... the patient does not know how to take... though we explain to him upon discharge... he doesn't understand..." (6E, Community Nurse)

Patient's preference was also highlighted by the participants. This is an important aspect to be considered, apart from the lack of medication knowledge, because some patients have a strong desire to stay in the hospital or refuse transferring to an old age home:

"The most challenging is the preference of the patient or his/her family... e.g., the patient's situation is worse after stroke... doesn't want to leave the hospital... and we don't have any regulation to discharge the patient." (6B, Medical Social Worker)

"Some patients don't want to live in an old age home... we know the patient must be readmitted if he stays at home... because he does not have any carer at home."(6E, Community Nurse)

#### 1.4 Social Factor

Participants highlighted the service availability issues in terms of waiting time, patient's affordability, and inadequate equipment. Following are some typical comments related to these issues:

*"The patients sometimes need to wait for 2*-*3 days for post-discharge support service... Also, there is no service available on weekends." (6B, Medical Social Worker)*

Participants further pointed out that unmatched need, transportation issue, and time gap were the most serious problems:

"Transportation is the biggest barrier to access the post-discharge support service...due to no transportation available, the patient cannot go to day center or clinic follow-up... Even though the patient pays, he cannot get the transportation service." (6F, Physiotherapist)

"For the referral to day care center, the patient has the referral letter from MSW upon discharge and is then assessed by the Department of Social & Welfare after discharge; thus, there is a time gap between the patient discharge and the service available." (6B, Medical Social Worker)

In addition, participants expressed there was poor multidisciplinary communication and coordination between hospital and community service provision:

"We are in the multidisciplinary team... really want more communication among us..." (6C, Medical Social Worker)

"If we find out some difficulties, we sometimes cannot find patient's medical chart because it is already sent to the record office... We cannot find the responsible person for answering the question. We really want direct dialogue..." (6E, Community Nurse)

### Theme 2: Suggestion on the important components for effective discharge planning

Various suggestions were provided by the participants regarding the barriers to discharge planning. Most of the participants agreed to have early screening to identify high-risk re-admitters using a simple screening tool. Having the screening tool installed in the information technology system would be beneficial because healthcare disciplines in different settings, e.g., acute hospital, rehabilitation hospital, and community-based service provision, would be alerted regarding the readmission risk of the patient. Implementing a standard screening procedure requires protocol and a policy-driven approach toward every staff member.

"I agree with others' suggestions that (once the patient is admitted to the hospital), we should perform the screening to identify high-risk readmitters, then each discipline will do his best to help... We also have a checklist to make sure everything has been done before the patient is discharged... Everything should be protocol-driven..." (3D, Doctor)

"We want a standardized (discharge planning) protocol, so everyone knows when to perform... even patients know clearly what the procedure is or if he would be involved" (6E, Community Nurse)

Participants further pointed out that the discharge planning should be a multidisciplinary approach, with clear roles for each healthcare discipline. A few roles were suggested, such as designated nurse or physician for discharge planning, clinical pharmacist for medication reconsideration, and trained volunteer for facilitation on psychosocial need:

"Once the patient is admitted to the hospital, we should let the patient know whom he can ask for help. Otherwise, the patient is confused with different party roles."(6E, Community Nurse)

"Now, a clinical pharmacist has been performing medication reconsideration for two years... we feel they can help a lot... we and patients know clearly the medication... Also, there is medication education for patients as well." (6D, Nurse)

"We have trained many volunteers and they are capable to help more especially on the patient's psychosocial need... we should make use of them, e.g., they can provide a brief orientation for each hospital admitter." (6E, Community Nurse)

Apart from the screening and manpower management, participants emphasized the provision of psychological support for patients, as well as education for managing the need of patient and carer:

"Patients' psychological problem is one of the major issues. We should pay more effort on the social support, then everything will be more smooth..." (6D, Nurse)

"Ninety-year-old lady falls and hopes to recover within 2 weeks... (5A, Medical Social Worker) Thus, we should communicate with the patient and her family early... we should give them the right information as early as possible... Otherwise, there will be an expectation gap between healthcare staff and patient." (5F, Physiotherapist)

With regard to community service management, participants highlighted the coordination between HA and community service provision. Allocation of more resources in the community service provision needs to be reviewed or improved:

"If more resources are allocated in the community setting which can enhance the pre-discharge support and decrease the readmission, it will decrease the cost on acute care and workload of healthcare staff in the acute setting. Thus, don't think the expensive community service is not worth it... it can decrease the cost in A&E and in-patient hospitalization." (6G, Occupational Therapist)

Some participants suggested changing physician's culture and/or enriching physician's training by emphasizing the psychosocial component:

"We don't worry about the clinical part, but we really worry about the social matters. The physicians are not mature enough to be aware of patient's social need... They should let a nurse to step in to help or a senior physician should play a role for guidance." (4E, Doctor)

A few participants expressed that, in some cases, family or carers were not available to take care of the patient at daytime, and the family cannot support the 24-hour domestic helper in terms of salary or living space. Thus, the participants suggested designating a daytime carer to provide round-the-clock care and supervision:

"Just like daytime domestic helper, she can stay with the patient... Most of elderly are very frail and need round-the-clock care... The government can allocate a pool of money to pay for the daytime domestic helper who can be the family member, then the family member can quit his/her job and take care of the elderly without affecting living cost..." (4B, Occupational Therapist)

Finally, majority of the participants suggested the discharge program should target all high-risk re-admitters without age limit. The follow up period of post discharge program should be flexible according to patient need. The group mentioned that the current IDSP should be more flexible and not limit follow up to only six months for selected high-risk cases. The participants agreed on the benefit of the IDSP on patient's health and readmission rate. Thus, they suggested IDSP could cover high-risk patients aged ≤ 65 years.

"Now, IDSP only serves geriatric patient... but some adult cases which do not reach age 60 are at high risk for readmission. They only receive CNS or GOPC support, it is not enough. IDSP is very good because it is a multidisciplinary approach and provides a platform for continual care and multidisciplinary communication. Since there is a time gap issue for community service, IDSP could bridge the gap." (2E, Occupational Therapist)

The summary of overall findings is shown in Table 2.

**Table 2:** Summary of important component of results

Current Practice
No standardized policy/protocol for discharge process
No standardized tool for facilitating the discharge process
Piece-meal approach in individual hospital
Discharge program targeting high risk readmission which is based on clinical judgment and varies across hospitals
Disease-specific discharge program for selected diseases

**Barriers to Discharge Planning**

***System Factor***
Lack of guideline or polices for the standardized discharge process/care pathway
Piece-meal program as pilot and issue of inflexibility of program
Pressure on bed availability
Poor medication system in hospital
Poor communication among healthcare disciplines
Issue of manpower shortage and management
Poor regulation of care quality in old age home
***Professional Factor***
Unclear role of each disciplines
Nurses not empowered to initiate discharge planning
Unclear or incomplete chart documentation
Low awareness on patient's social needs
***Patient Factor***
Lack of knowledge of medication/treatment
Mis-concept of hospital discharge
***Social Factor***
Issue of services availability - waiting time, affordability, equipment loan
Issue of un-match needs of patients - transportation, time gap of service availability and hospital discharge
Poor communication/coordination between hospital and community service provision

**Suggestion on Importance Components for Effective Discharge Planning**

Standard screening tools to identify high risk readmission case with protocol approach and policy-driven
Discharge planning with multidisciplinary approach
Clear role of each multidisciplinary identified in the discharge planning
Designed nurse/physician for discharge planning as contact point
Clinical pharmacist for medication reconsideration
Trained volunteer for identification/facilitation on patient's psychosocial needs
Effective manpower management
Patient education: medication/treatment, concept of discharge process
Coordination between Hospital Authority/hospitals and community service provision
Enhance training/education on patients' psychosocial needs for physicians
Home carer support program to facilitate transition period from hospital discharge to home

## Discussion

In the present study, the views of different healthcare professionals on current discharge planning and barriers encountered in the aspects of system, healthcare professional, patient, and society were explored. Participants highlighted that there was no systematic hospital-wide discharge planning and policy-driven approach in public health sector; and its potential barriers with regard to factor of system, healthcare professionals, patients, and social. The findings help provide important insights into the development of effective discharge planning so as to improve the quality of in-patient care.

Many opinions regarding the present discharge planning process were provided by the healthcare professionals currently responsible for coordinating the discharge planning process. Majority of the participants thought that the present discharge program was a piecemeal approach. There was no standardized and policy-driven discharge planning protocol, which was agreed to be important in facilitating the discharge planning process to decrease unnecessary hospital readmission. In the UK, US, and Australia, a standardized discharge planning protocol is being launched as a policy-driven guideline for healthcare staff to execute [19,20,29]. The UK further specified that discharge planning should be classified as simple or complex discharge upon the point of patient admission [29]. At least 80% of patients belong to simple discharge, whereas the rest belong to complex discharge. The latter requires a multidisciplinary team to coordinate the services and design a care plan with all the parties concerned [29]. The classification of complex and simple discharge could be helpful to design the discharge planning process. Overall, the participants recognized that a policy-driven discharge program was necessary to establish thorough and effective discharge planning.

As identified in literature, many factors affect discharge planning. The barriers encountered by the participants are mainly categorized into four aspects: system, healthcare professionals, patients, and social. The discussion of potential system factor focused on premature discharge due to the pressure of bed unavailability, manpower management, and lack of communication among different healthcare professionals and with the community service provision. In line with other studies, lack of staff, poor communication, and the pressure to discharge patients in a timely manner may contribute to inappropriate discharge [30,31]. The findings, consistent with other literature, suggest the need to examine staffing patterns and discharge planning procedures [30]. Participants further suggested that a standardized and hospital-wide discharge planning protocol taking into account a multidisciplinary approach is critical. The role of each member of the healthcare team is also clearly identified as important [32]. The NHS report (2004) highlighted that the roles of a multidisciplinary team have to be clarified and agreed as to who, how, and when the expected discharge date will be based on anticipated length of stay. This is assessed and documented, communicated to the patient and carer, and reviewed on a daily basis [29]. A clear role of each member of the healthcare team in relation to discharge planning is beneficial to patients as well [33]. Moreover, participants suggested having a standard discharge program covering all patients who have high-risk readmission rate. Thus, further research and study are required to improve the discharge planning system by having a standard tool for screening, assessment, and pre-discharge checklist with clear guidelines/protocol. Poor communication has always been an issue to effective discharge from hospitals; therefore, the communication and acceptability for study of a protocol-driven measure is required in future study. Skeet (1975) highlighted this problem over 20 years ago, as well as the need for good communication between acute and sub-acute service provision [34]. This 20-year-old problem was again emphasized by a number of studies [35,36]. This present study supports that the poor communication issue still exists. Promoting the use of information technology, as suggested by the participants, could help overcome this problem in the future, and has been addressed in other literature [37].

Another barrier is the healthcare professional factor. Awareness and knowledge of patient's psychosocial need were inadequate, which further suggests that a comprehensive assessment tool is required. Moreover, a change in focus is needed from disease management as the central measure of successful discharge planning to a communicative, ethical approach that promotes quality of life for both the patient and the carers involved in the discharge process [38]. In Australia, the comprehensive discharge strategy identified assessment of patient physiological, psychological, social, and cultural needs as equally important components in the effective discharge process [19]. A thorough and individualized patient assessment is required to ascertain these needs. In addition, healthcare workers agreed that good documentation is the most beneficial item. However, majority of the participants expressed that not everyone had the practice or no planning at all. Research revealed that some healthcare professionals do not regard paperwork as having the same status as patient care [39,40]. Thus, healthcare professionals may not be aware of the importance of good documentation and having adequate knowledge of discharge planning. Improving documentation among healthcare professionals can involve inter-professional education and health policy.

A patient factor which was a potential barrier to discharge planning, as pointed out by the majority of participants, was the lack of patient knowledge of medication treatments. As mentioned above, communication between patients and healthcare professionals regarding patient needs after discharge is a critical component of effective discharge planning and continuity of care. Tailored information for each patient should be prepared because of the differences in communication ability of each patient. Patients normally do not have the vocabulary and skills to speak with healthcare workers [41]. Patient preference was another barrier to discharge planning. A patient could have adequate discharge information and post-discharge care requirement (such as medication reconciliation). However, noting and understanding patient's potential role and preference through effective communication is also important [42]. The lack of communication also influences the carer's ability to manage the patient at home. Such a finding would indicate that the improvement of communication skill is needed in both healthcare professionals, patients and carer [43,44].

Service and time gap of post-discharge support were also highlighted as major barriers in the social aspect, reflecting the presence of issues in communication, resources allocation, and matching patient's needs. The suggestion put forward by respondents centered on the coordination between the hospital and the community service provision, as well as the review of resource allocation in the community service provision. Again, there is a need to re-educate both sectors as to what information is required. The format in which information is required would facilitate better understanding and smoother transfer of patients from hospital to home/community [40]. A collaboration approach is needed in the acute care and sub-acute care provisions in the whole healthcare system.

This present study has two limitations. The focus group involved healthcare professionals with at least 10 years because they were more experienced to share with regard to the topic. However, a mixed focus group is more likely to raise issues of multidisciplinary working; thus, we may lose voice from junior healthcare professionals. Also, only service providers from different healthcare disciplines and positions, including frontline and management, were recruited. Thus, the service user from the patient perspective was not included. Exploration of the unmet clinical, educational, and psychosocial needs from the patient's perspective is needed in future research.

## Conclusions

Effective discharge planning requires capacity planning, performance review, hospital discharge policies, and healthcare providers/stakeholders agreements. There is clear evidence and wide agreement among healthcare providers/stakeholders that a standardized and policy-driven protocol was important to an effective discharge planning. This study has provided the important message that communication between health and social care professionals, between healthcare professionals and patients, and among healthcare professionals should also be improved and emphasized. The collaboration between different providers/healthcare professionals, between acute and rehabilitation/extend care, between hospital and community service sector, and between clinical and psychosocial sectors needs to be strengthened.

## Competing interests

The authors declare that they have no competing interests.

## Authors' contributions

All authors were involved in the design of the project and the survey tool and carried out the study. In addition, all team members provide assistance with data transcription and performed them coding and identification. The first draft of this article was composed by ELYW and was revised critically by all authors. All authors have approved the final version of the manuscript.

## Pre-publication history

The pre-publication history for this paper can be accessed here:

http://www.biomedcentral.com/1472-6963/11/242/prepub
